# Expansion of the phenotypic spectrum and description of molecular findings in a cohort of patients with oculocutaneous mosaic RASopathies

**DOI:** 10.1002/mgg3.625

**Published:** 2019-03-19

**Authors:** Oscar F. Chacon‐Camacho, Daniel Lopez‐Moreno, Martha A. Morales‐Sanchez, Enriqueta Hofmann, Michelle Pacheco‐Quito, Ilse Wieland, Vianney Cortes‐Gonzalez, Cristina Villanueva‐Mendoza, Martin Zenker, Juan Carlos Zenteno

**Affiliations:** ^1^ Department of Genetics Institute of Ophthalmology “Conde de Valenciana” Mexico City Mexico; ^2^ “Dr. Ladislao de la Pascua” Dermatologic Center Mexico City Mexico; ^3^ Department of Glaucoma Institute of Ophthalmology “Conde de Valenciana” Mexico City Mexico; ^4^ Institute of Human Genetics, University Hospital Magdeburg Germany; ^5^ Department of Genetics Hospital "Dr. Luis Sanchez Bulnes", Asociación para Evitar la Ceguera en México Mexico City Mexico; ^6^ Department of Biochemistry, Faculty of Medicine UNAM Mexico City Mexico

**Keywords:** encephalo‐cranio‐cutaneous lipomatosis, FGFR1, KRAS, mosaicism, mucocutaneous hyperpigmentation, oculoectodermal syndrome, RASopathies, Schimmelpenning‐Fuerstein‐Mims syndrome

## Abstract

**Background:**

Postzygotic *KRAS*, *HRAS*, *NRAS*, and *FGFR1* mutations result in a group of mosaic RASopathies characterized by related developmental anomalies in eye, skin, heart, and brain. These oculocutaneous disorders include oculoectodermal syndrome (OES) encephalo‐cranio‐cutaneous lipomatosis (ECCL), and Schimmelpenning‐Feuerstein‐Mims syndrome (SFMS). Here, we report the results of the clinical and molecular characterization of a novel cohort of patients with oculocutaneous mosaic RASopathies.

**Methods:**

Two OES, two ECCL, and two SFMS patients were ascertained in the study. In addition, two subjects with unilateral isolated epibulbar dermoids were also enrolled. Molecular analysis included PCR amplification and Sanger sequencing of *KRAS*, *HRAS*, *NRAS*, and *FGFR1* genes in DNA obtained from biopsies (skin/epibulbar dermoids), buccal mucosa, and blood leukocytes. Massive parallel sequencing was employed in two cases with low‐level mosaicism.

**Results:**

In DNA from biopsies, mosaicism for pathogenic variants, including KRAS p.Ala146Thr in two OES subjects, FGFR1 p.Asn546Lys and KRAS p.Ala146Val in ECCL patients, and KRAS p.Gly12Asp in both SFMS patients, was demonstrated. No mutations were shown in DNA from conjunctival lesions in two subjects with isolated epibubar dermoids.

**Conclusion:**

Our study allowed the expansion of the clinical spectrum of mosaic RASopathies and supports that mosaicism for recurrent mutations in *KRAS *and *FGFR1 *is a commonly involved mechanism in these rare oculocutaneous anomalies.

## 1 INTRODUCTION

Phenotypic variability of human genetic diseases can occasionally arise from genetic mosaicism, which is defined by the presence of two or more populations of cells with distinct genotypes in one individual arising from a single fertilized egg (De, 2011). Mosaicism is caused by postzygotic mutation(s) and the extent of affected tissues and clinical manifestations will depend on the stage of development at which the mutational event occurs (Cohen, Wilson, Trinh, & Ye, 2015). When the DNA variation occurs somatically, the condition is termed somatic mosaicism and can be suspected in subjects with body asymmetries or altered body pigmentation patterns (Cohen et al., 2015; Spinner & Conlin, 2014). Recently, pathogenic variants in a number of known proto‐oncogenes, which are assumed to be lethal when occurring in the germline, have been shown to occur as segmental mosaicism in a variety of clinical syndromes. These disorders include congenital lipomatous overgrowth, vascular malformations, and epidermal nevi (CLOVE) [OMIM #612918] (Kurek et al., 2012), Mafucci [OMIM %614569] (Amary et al., 2011), Proteus [OMIM #176920] (Lindhurst et al., 2011), and Sturge‐Weber [OMIM #185300] (Shirley et al., 2013) syndromes, among others. More specifically, mosaicism for mutations in genes involved in the Ras/MAPK signaling pathways is the cause of a group of disorders termed mosaic RASopathies, which include keratinocytic epidermal nevus syndrome (Bourdeaut et al., 2010), sebaceous nevus syndrome (Groesser et al., 2012), phakomatosis pigmentokeratotica (Groesser et al., 2013), nevus spilus (Sarin, McNiff, Kwok, Kim, & Khavari, 2014) and neurocutaneous melanosis/congenital giant melanocytic nevi [OMIM #137550] (Kinsler et al., 2013), wooly hair nevus [OMIM #169200] (Levinsohn et al., 2014), and cutaneous‐skeletal hypophosphatemia syndrome (Lim et al., 2014). Three additional syndromes with overlapping clinical findings in eye, heart, skin, and hair have been recently shown to result from somatic mosaicism for mutations in the genes involved in the Ras pathway, including *KRAS* [OMIM *190070], *HRAS* [OMIM *190020], *NRAS* [OMIM *164790], and *FGFR1* [OMIM *136350]. These entities are the oculoectodermal (OES, OMIM %600268) and Schimmelpenning‐Feuerstein‐Mims syndromes (SFMS, OMIM #163200), as well as encephalocraniocutaneous lipomatosis (ECCL, OMIM #613001), oculocutaneous disorders exhibiting pleiotropic anomalies that include scalp lesions, epilepsy, epibulbar dermoids, cloudy cornea, eyelid coloboma, coarctation of the aorta, and skin pigmentation abnormalities (Boppudi et al., 2016; Groesser et al., 2012; Kuroda et al., 2015; Peacock et al., 2015). Somatic mutations causing oculocutaneous mosaic RASopathies are recurrent and have been identified at *KRAS *codons 13, 19, and 146 in OES patients (4 subjects reported to date) (Boppudi et al., 2016; Peacock et al., 2015), *KRAS *codon 146 and *FGFR1 *codons 546 and 656 in ECCL (six reported patients) (Bennett et al., 2016; Boppudi et al., 2016), and *HRAS *codon 13 (2 patients), *KRAS* codon 12 (3 subjects), and *NRAS* codon 61 (1 subject) in individuals with a SFMS diagnosis (Groesser et al., 2012; Igawa et al., 2016; Kuroda et al., 2015; Lihua, Feng, Shanshan, Jialu, & Kewen, 2017; Sun et al., 2013; Wang, Qian, Zhang, & Zhou, 2015). Such pathogenic mutations also confer an elevated risk for developing a variety of tumors commonly associated with these oculocutaneos disorders (Aslan et al., 2014; Eisen & Michael^2009^, ^2009^; ^2011^; Kocak, Yarar, & Carman, 2016; Moog, 2009; Peacock et al., 2015; Toriello et al., 1999; Valera et al., 2012).

In order to expand the knowledge on the clinical and genetic features of mosaic RASopathies, we describe here the phenotypic and molecular characteristics of a cohort of Mexican patients with a clinical diagnosis of these disorders. Additionally, two patients with unilateral isolated epibulbar dermoid were also genotyped for the search of somatic mutations in Ras/MAPK signaling pathway genes. Our study allowed the expansion of the clinical spectrum of mosaic RASopathies and supports that somatic mosaicism for recurrent mutations in *KRAS *and *FGFR1 *is a commonly involved mechanism in these rare oculocutaneous developmental anomalies.

## 2 MATERIALS AND METHODS

### 2.1 Patients

The study was approved by local Institutional Review Board and Ethics committees and was conducted according to the principles expressed in the Declaration of Helsinki. Parents of patients signed a written informed consent for clinical and molecular studies and for tissue biopsies.

Six unrelated patients with suspected oculocutaneous somatic mosaicism were included in the study. All of them had a complete ophthalmologic and dermatologic examination. When possible, imaging studies such as cranial computed tomography and magnetic resonance imaging, ocular ecography, echocardiogram, and abdominal ultrasonography were performed. In addition, two patients with a diagnosis of isolated unilateral epibulbar dermoids were enrolled in the study.

For patient #1 and #2, small biopsies were obtained from aplasia cutis lesions on the head and from hyperpigmented skin regions on the neck. A biopsy of a nevus psiloliparus was obtained in patient #3 while biopsies from sebaceous nevi from patients #4 and #5 were obtained. Patients #6–8 underwent biopsies from epibulbar dermoids. In all cases, tissue samples were used for DNA isolation. In addition, peripheral leukocytes and buccal mucosa cells were employed for DNA isolation in all six patients (#1–6) with syndromic entities.

### 2.2 Molecular analysis

Tubes containing skin biopsies were placed in a TissueLyser Adapter Set for disruption and homogenization of tissues, following the manufacturer's recommendations (TissueLyser II system, Qiagen, Hilden, Germany). Genomic DNA from biopsy tissues, peripheral blood leukocytes, and buccal mucosa cells was isolated using the QIAamp DNA Mini Kit (Qiagen, Hilden, Germany), and Gentra Puregene Buccal Cell Kit (Qiagen, Hilden, Germany), following the manufacturer´s instructions. The coding regions of *KRAS*, *HRAS, NRAS*, and *FGFR1 *genes and their adjacent intronic sequences were amplified by PCR using pairs of primers corresponding to Ensembl reference sequences (*KRAS, *ENST00000311936.7; *HRAS, *ENST00000451590.5; *NRAS, *ENST00000369535.4; *FGFR1, *ENST00000447712.6). Direct automated sequencing of exons of *KRAS*, *HRAS, NRAS, *and *FGFR1* was performed with the BigDye Terminator 3.1 Cycle Sequencing kit (Applied Biosystems, Foster City, CA). All samples were analyzed either on an ABI 3130 or 3500xl Genetic Analyzer (Applied Biosystems) and sequences were compared against the respective reference sequences. The mosaicism level of the identified variants was estimated by comparing the area under the curve of electropherograms for wildtype and mutant peaks using the Mutation Surveyor software, as previously reported (Minton, Flanagan, & Ellard, 2011).

In patients #3 and #6, massive parallel sequencing was applied using a TruSeq Custom Amplicon Low Input Library Prep Kit (Illumina) as recommended by the supplier for DNA enrichment. Massive sequencing was performed on a next‐generation sequencing platform (MiSeq System, Illumina). The target regions included hotspots for mosaic mutations in the genes *KRAS*, *HRAS*, *NRAS*, *FGFR1‐3*, among others. Target amplicons showed a read depth between 1,000 and 5,000 reads. Sequence data were analyzed by Variant Studio Data Analysis Software 3.0 (Illumina). Application of this procedure achieved a detection limit as low as 1% for mutation hot spots.

## 3 RESULTS

### 3.1 Clinical reports

#### 3.1.1 Patient #1

**Figure 1:**
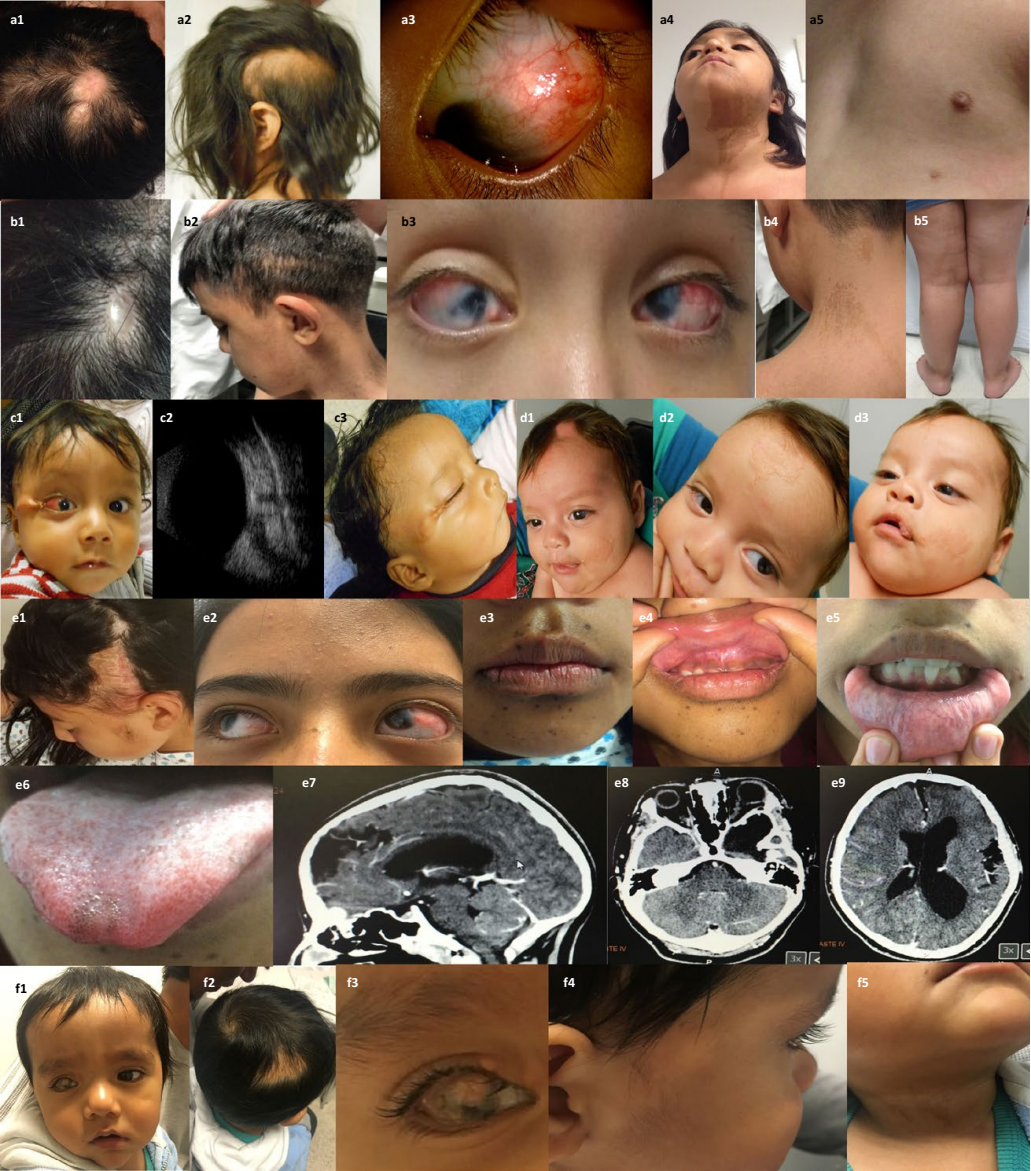
Clinical characteristics of patients with oculocutaneous mosaic RASopathies. (a) OES patient #1. (a1) Two aplasia cutis verticis lesions; (a2) Left parietal alopecia; (a3) Epibulbar dermoid on the left eye; (a4) Hyperpigmentation on left mandibular region and left side of the neck; (a5) Polythelia. (b) OES patient #2. (b1) Aplasia cutis verticis; (b2) Temporal‐parietal alopecia; (b3) Bilateral epibulbar dermoid; (b4) Cutaneous hyperpigmentation on the posterior neck; (b5) Lymphedema of left limb. (c) ECCL patient #3. (c1) Nevus psiloliparus on right zygomatic region; (c2) Right optic nerve coloboma; (c3) Alopecia frontal, parietal, and nevus psiloliparus on the temporal‐parietal regions. (d) SFMS patient #4. (d1–3) Nevus sebaceous on the left parietal‐frontal‐maxilar, and upper lip regions. (e) SFMS patient #5. (e1) Nevus sebaceous on the left temporal‐parietal regions; (e2) Epibulbar dermoid in left eye; (e3) Hyperpigmentation around the mouth, and multiple hyperpigmented nevi inside the cutaneous pigmentation lesion are evident; (e4–6) Hyperpigmentation of upper and lower gingival mucosa as well as in the tip of the tongue. (e7) Cranial computed tomography evidenced a left temporal arachnoid cyst, (e8) a left eye retinal coloboma, and (e9) hemimegalencephaly; (f) ECCL patient #6. (f1) Facial appearance. Brachycephaly was noted; (f2) Alopecia in right parietal region; (f3) Right eye: epibulbar dermoid and small nodular tag in the upper lid; (f4) Skin hypoplasia in the right zygomatic and preauricular regions; (f5) Skin linear hyperpigmentation in the neck

A 4‐year‐old girl was referred due to a congenital anomaly in her left eye and was clinically diagnosed with OES. She was the third child of unrelated, healthy parents and her family history was uneventful. Her mother did not receive prenatal care but denied exposure to teratogenic agents. The proband was born at 41 weeks of gestation via spontaneous vaginal delivery and her birth length was 46 cm (3–10th centile), weight 2,700 g (10–25th centile), and Apgar scores of 7/8. Psychomotor development was normal. At birth, a left eye conjunctival mass and scalp lesions (Figure 1—a1–3) were noted. At present examination, she exhibited left parietal alopecia and two areas of aplasia cutis near to vertex capitis (Figure 1—a1,2). Inspection of the left eye revealed a subconjunctival temporal tissue mass extending to the corneal limbus and temporal cornea, with no apparent interference with the visual axis (Figure 1—a3). On funduscopic examination, optic nerve excavations of 50% and 80% in right and left eyes, respectively, were observed. In addition, bilateral epicanthal folds, downslating palpebral fissures, low‐set ears, broad nasal tip and bridge, downturned corners of the mouth, and left facial hemihyperplasia were also seen. Skin examination disclosed hyperpigmented lesions on the left side to the body, spreading‐out over the left hemimandible (Figure 1—a4) and zygomatic region, left side of the neck, and downward following the middle line involving the suprasternal notch, upper back, and left arm. Left polythelia (Figure 1—a5) and hypertrichosis were also evident. Echocardiogram evidenced atrial septal defect (ASD) and persistent ductus arteriosus (PDA) (Table 1). Cranial computed tomography did not demonstrate brain anomalies. No neurological symptoms were present.

**Table 1:** Clinical and molecular characteristics of Mexican subjects with mosaic RASopathies

Patient #	Gender	Age at evaluation	Gene/pathogenic variant	Frequency of mutation (DNA isolated from)	Epibulbar dermoid	Cardiac anomalies	Alopecia	Aplasia Cutis	Cutaneous Hyper‐pigmentation	Nevus Psiloli‐parus	Nevus Sebaceous	Central Nervous System anomalies	Novel clinical findings	Clinical diagnosis
1	F	4 years	*KRAS* c.436G>A p.Ala146Thr	28% (AC) NA (HP)	+Unilat.	+ASD PDA	+	+	+	−	−	−	Facial asymmetry, left body hemihypertrophy, supernummerary nipple, ASD +PDA	OES
2	M	12 years	*KRAS *c.437C>T p. Ala146Val	27% (AC) 26% (HP)	+Bilat.	+HC	+	+	+	−	−	−	Microcornea, nystagmus, short neck, hypertrophic cardiomyopathy	OES
3	M	6 months	*FGFR1 *c.1638C>A p.Asn546Lys	36% (NP)	+Unilat.	−	+	−	−	+	−		−	ECCL
4	M	4 months	*KRAS *c.35G>A p.Gly12Asp	24.3% (LNS)	+Unilat.	−	+	−	−	−	+	+	Bilateral hearing loss	SFMS
5	F	12 years	*KRAS *c.35G>A p.Gly12Asp	52% (HNS) 13% (HP)	+Unilat.	−	−	+	+	−	+	+	hyperpigmentation at peribuccal region, gums, and tongue tip	SFMS
6	M	10 months	*KRAS *c.437C>T p.Ala146Val	<10% (ED)	+Unilat.	−	+	−	+	−	−	+	−	ECCL
7	F	14 years	(−)	−(ED)	+Unilat.	−	−	−	−	−	−	−	−	IED
8	M	2 years	(−)	−(ED)	+Unilat.	−	−	−	−	−	−	−	−	IED

Note

+: present; −: absent.

AC: aplasia cutis; ASD: atrial septal defect; Bilat.: bilateral; ECCL: encephalo‐cranio‐cutaneous lipomatosis; ED: epibulbar dermoid; FA: facial asymmetry; F: female; HC: hypertrophic cardiomyopathy; HNS: head nevus sebaceous; HP: hyperpigmented lesion; I: isolated; IED: isolated epibulbar dermoid; L: left; LNS: lip nevus sebaceous; M: male; NA: no PCR amplification; NP: nevus psiloliparus; OES: oculoectodermal syndrome; PDA: persistent ductus arteriosus; R: right; SFMS: Schimmelpenning‐Feuerstein‐Mims syndrome; Unilat.: unilateral.

#### 3.1.2 Patient #2

A 12‐year‐old male was clinically diagnosed with OES. He is the fifth child of healthy, nonconsanguineous parents and was referred due to bilateral epibulbar dermoids. All of his living siblings were healthy and his father had a history of scapular lipectomy for cutaneous lipomatosis. At birth he had a weight of 4,475 g (>97th centile), length of 51 cm (25–50th centile), and Apgar scores of 8/9. Examination at the age of 11 months disclosed bilateral epibulbar dermoids, microcornea, endotropia, and nystagmus. The visual axes were involved by dermoid tissue extension to both corneas. Cardiovascular examination and electrocardiogram revealed hypertrophic cardiomyopathy at 4 years of age. At present examination, several somatic anomalies were evident including frontal bossing, aplasia cutis verticis of 1 × 1 cm (Figure 1—b1), parietal alopecia (Figure 1—b2), bilateral epibulbar dermoids (Figure 1—b3), bilateral epicanthal folds, short neck, umbilical scar due to umbilical hernia repair surgery, asymmetry of lower limbs due to lymphedema of left limb, and shortening (1 cm) of the left lower limb (Figure 1—b5). Cutaneous hyperpigmentation affecting the left side of the body was noted particularly at the left parotid region, spreading‐out on the left anterior, posterior and lateral neck (Figure 1—b4), as well as in left dorsal and lumbar regions. Hyperpigmented skin lesions were also noted on abdomen as well as on the posterior region of the left thigh. Abdominal ultrasonography and doppler ultrasound of lower limbs excluded visceral or vascular anomalies. No neurological symptoms were present.

#### 3.1.3 Patient #3

This 6‐month‐old boy exhibited a round and soft mass of 4 × 2 cms located at the right zygomatic and conjunctival areas and was clinically diagnosed with ECCL. The propositus is the second child of nonconsanguineous, healthy parents. His mother received regular prenatal care and was not exposed to teratogenic agents. He was born by spontaneous vaginal delivery and his birth length was 43 cm (<3rd centile), and weight 2,045 g (<3rd centile). He exhibited alopecia in the anterior midline, parietal, and temporal areas as well as a smooth hairless fatty nevus (nevus psiloliparus) at the right zygomatic and temporo‐parietal regions (Figure 1—c1,3). Two small nodular skin tags outside of the outer canthus and a left conjuctival lesion suggestive of choristoma were also identified. Ocular ultrasound demonstrated right optic nerve coloboma (Figure 1—c2). No other anomalies were found. No central nervous system imagenologic studies were carried out. At present, no neurological anomalies were present in this patient.

#### 3.1.4 Patient #4

This is a 2‐year‐old male referred due to cutaneous sebaceous lesions, epilepsy, and left eye leucoma. He was clinically diagnosed with SFMS. He is the second child of consanguineous (first cousin), healthy parents and was born at term by cesarean section with a birth length of 51 cm (25–50th centile), weight 4,100 g (75th centile), and Apgar score of 6/7. Tonic‐clonic convulsions developed at 20 days of age. His examination demonstrated absence of hair in frontal midline and left parietal areas. Two large oval orange‐yellowish nevus sebaceous lesions were observed in the forehead and left parietal region (Figure 1—d1,2). Other patches were noted on the left cheek and left region of the upper lip (Figure 1—d3). A linear sebaceous lesion was seen over the nose tip whereas a temporal bulbar dermoid was observed in the left eye. Wide nasal bridge and anteverted nares were also noted. Ocular ecography showed left optic nerve coloboma while auditory evoked potentials identified bilateral hearing loss. A cranial computed tomography demonstrated cortical atrophy, interhemispheric asymmetry, left polymicrogyria, and ventriculomegaly.

#### 3.1.5 Patient #5

A 12‐years‐old female who was evaluated due to a sebaceous nevus on the head received a clinical diagnosis of SFMS. She was the third child of a nonconsanguineous and healthy couple. Her mother received adequate prenatal care and had no exposure to teratogenic agents. Her birth measurements were not provided but were reported as normal. A biopsy of the nevus sebaceous lesion was performed at one year of age but no results were provided to the family. At present examination, she exhibited a linear nevus sebaceous at the left parotid, temporal, and parietal regions (4 × 12 cm) (Figure 1—e1), which extended to a round aplasia cutis lesion (1.5 × 1.5 cm) in the left parietal area (Figure 1—e1). An epibulbar dermoid was present in the left eye (Figure 1—e2). Skin hyperpigmentation was observed in the left frontal region (8 × 3 cm) (Figure 1—e2), around the mouth (Figure 1—e3), in both upper and lower gingival mucosa (Figure 1—e4,5), and in the tip of the tongue (Figure 1—e6). Additional café‐au‐lait spots were present through her body while multiple hyperpigmentated nevi were noted around the mouth (Figure 1—e3,4). Cranial computed tomography evidenced a left temporal arachnoid cyst (Figure 1—e7), hemimegalencephaly (Figure 1—e9), and left eye retinal coloboma (Figure 1—e8). Despite her brain anomalies, she was free of neurological symptoms at present examination.

#### 3.1.6 Patient #6

This is a 10 months‐old male who was seen due to alopecia and epibulbar dermoid in the right eye. He was the second child of consanguineous and healthy parents. His mother received regular prenatal care and was not exposed to teratogenic agents. He was born at term by cesarean section and his birth length was 50 cm (50th centile), weight 3,500 g (50th centile) and Apgar score of 6/7. His examination revealed brachycephaly, focal alopecia in the right parietal area, and a right upper lid small nodular skin tag. An epibulbar dermoid expanding to right cornea was present. In addition, focal skin hypoplasia in the right zygomatic area, which extended to the temporal and preauricular regions was observed. Linear hyperpigmentation on the right lateral neck and on the cervical back were also identified. Right cryptorchidism was present. MR disclosed arachnoid cyst in right temporal region as well as right cortical atrophy. No neurological manifestations were present.

#### 3.1.7 Patients #7 and #8

These subjects were a boy aged 2 years and a girl aged 14 years, both exhibiting unilateral isolated epibulbar dermoids. No systemic features or neurological symptoms were identified in them and their family history was negative for congenital anomalies. Biopsy of dermoid tissue was performed during ocular surgery in both patients.

### 3.2 Molecular results

**Figure 2:**
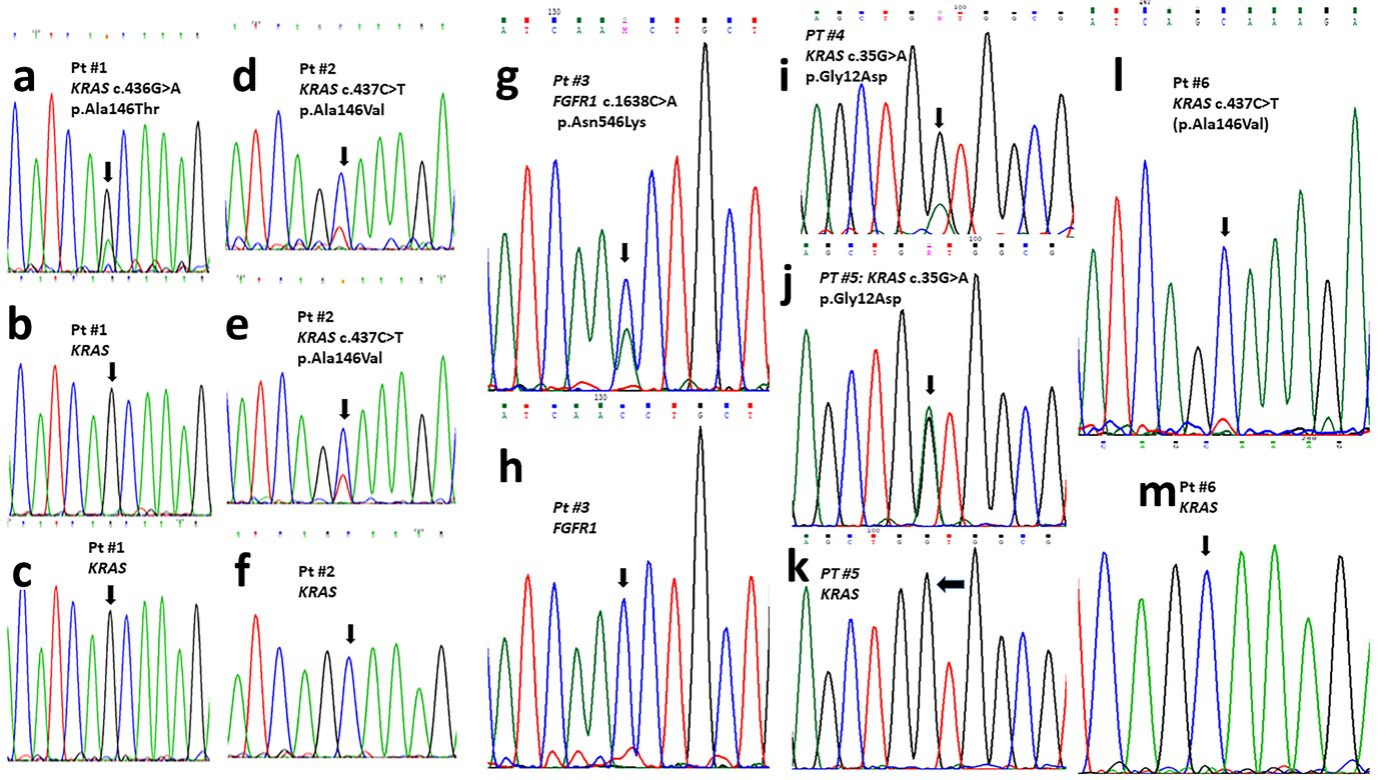
Molecular analysis of RAS‐related genes. Patient #1 (a–c). (a) *KRAS* partial sequence showing the c.436G>A (p.Ala146Thr) mutation in DNA from the aplasia cutis lesion; This variant was not identified in DNA from buccal mucosa (b) or peripheral leukocytes (c). Patient #2 (d–f). (d,e). *KRAS* partial nucleotidic sequence showing c.437C>T (p.Ala146Val) mutation in DNA obtained from the aplasia cutis (d) and hyperpigmented skin (e) lesions. This variant was not identified in DNA from buccal mucosa or leukocytes (f). Patient #3 *FGFR1* partial nucleotidic sequence showing the pathogenic c.1638C>A (p.Asn546Lys) variant identified in DNA from nevus psiloliparus (g). This variant was not identified in DNA from buccal mucosa or peripheral leukocytes (h). Patient #4 (i), patient #5 (j). *KRAS* partial DNA sequence showing the pathogenic variant c.35G>A (p.Gly12Asp) in DNA from nevus sebaceous of parietal region (i), and upper lip (j). This variant was not identified in DNA from buccal mucosa or blood leukocytes (k, only patient #5 sequence is shown)). Patient #6 (l, m). *KRAS* partial nucleotide sequence showing the c.437C>T (p.Ala146Val) mutation (l) identified in DNA from an epibulbar dermoid. This variant was not identified in DNA from buccal mucosa (m) or blood leukocytes

No pathogenic variants in the *KRAS, NRAS,* *HRAS*, and *FGFR1 *genes were identified by Sanger sequencing in DNA isolated from blood leukocytes or buccal cells from any subject. However, different levels of mosaicism for specific mutations were demonstrated in DNA obtained from biopsies of skin lesions or epibulbar dermoids (Table 1). Five subjects exhibited *KRAS *mutations, including 2 OES patients with pathogenic variants at codon 146 (exon 4), c.436G>A (p.Ala146Thr) (patient #1, Figure 2a–c), and c.437C>T (p.Ala146Val) (patient #2, Figure 2d–f), respectively; two SFMS patients with an identical c.35G>A (p.Gly12Asp) *KRAS *variant in exon 2 (patients #4 and #5, Figure 2i–k; Table 2), and one ECCL patient carrying the c.437C>T (p.Ala146Val) mutation in *KRAS* (patient #6, Figure 2l). Patient #3 with a ECCL diagnosis was negative for mutations in *KRAS, HRAS, *and *NRAS* in DNA from skin biopsy when analyzed by Sanger sequencing; however subsequent next generation sequencing analysis allowed the identification of a c.1638C>A (p.Asn546Lys) variant in *FGFR1 *which was barely demonstrable by additional Sanger sequencing (Figure 2g,h; Table 1). According with the Mutation Surveyor software, the percentage of cells carrying these mutated alleles ranged from 13% to 52% (Table 1). No mutations in *KRAS, NRAS, HRAS* and *FGFR1* genes were demonstrated in DNA obtained from blood leukocytes or epibulbar dermoids from patients #7 and #8 with isolated epibulbar dermoids.

**Table 2:** Postzygotic pathogenic variants in patients with Schimmelpenning‐Feuerstein‐Mims syndrome reported in the literature

Patients #	Gene	Mutation	Reference
1	*HRAS*	c.37G>C; p.Gly13Arg	Groesser et al. (2012)
2	*KRAS*	c.35G>A; p.Gly12Asp	Groesser et al. (2012)
3	*HRAS*	c.37G>C; p.Gly13Arg	Sun et al. (2013)
4	*NRAS*	c.182A>G; p.Gln61Arg	Kuroda et al. (2015)
5	*KRAS*	c.35G>A; p.Gly12Asp	Wang et al. (2015)
6	*KRAS*	c.34G>T; p.Gly12Cys	Igawa et al. (2016)
7	*KRAS*	c.35G>A; p.Gly12Asp	Lihua et al. (2017)
8	*KRAS*	c.35G>A; p.Gly12Asp	Present study
9	*KRAS*	c.35G>A; p.Gly12Asp	Present study

## 4 DISCUSSION

Until recently, somatic cutaneous mosaicism was considered as a rare event with little or any clinical consequences. Recent genomic studies have recognized mutational somatic mosaicism in skin lesions with abnormal pigmentation, especially in those following the Blaschko lines (Amary et al., 2011; Groesser et al., 2012; Kurek et al., 2012; Peacock et al., 2015; Rivière et al., 2012; Shirley et al., 2013; Weinstein et al., 1991). The OES, ECCL, and SFMS syndromes are a group of oculocutaneous developmental mosaic RASopathies which exhibit common anomalies including focal scalp lesions, linear cutaneous hyperpigmentation, arachnoid cyst, and cardiac defects (Ardinger, Horii, & Begleiter, 2007; Ernst, Quinn, & Alawi, 2007; Moog, 2009). At the molecular level, these entities are caused by somatic mosaicism for mutations in genes encoding components of the RAS signaling pathway (Bennett et al., 2016; Boppudi et al., 2016; Groesser et al., 2012; Peacock et al., 2015). OES is a very rare disease characterized for a consistent combination of scalp lesions and epibulbar dermoids (Toriello, Lacassie, Droste, & Higgins, 1993). Aplasia cutis congenita is the most frequent skin finding and other body structures are commonly involved, including central nervous system (arachnoid cyst, dilatation of ventricules) (Gardner & Viljoen, 1994; Martin, Lockspieler, & Slavotinek, 2007), bones (bone cyst) (Toriello et al., 1999), urogenital tract (bladder exstrophy, epispadias, bladder rhabdomyosarcoma) (Lees, Taylor, Atherton, & Reardon, 2000), cardiovascular system (aortic coarctation, Moyamoya disease) (Horev, Lees, Anteby, Gomori, & Ben‐Neriah, 2011; Toriello et al., 1993). In our series, patients #1 and #2 received a clinical diagnosis of OES as they exhibited aplasia cutis and epibulbar dermoids, cardinal features of the disease. Of note, patient #1 presented with some features rarely reported in OES as facial asymmetry (Martin et al., 2007), glaucoma (Federici et al., 2004), lower limb lymphedema (Horev et al., 2011), ASD (Toriello et al., 1993), and PDA. Our data support that these anomalies can be considered as part of the disorder rather than coincidental findings in previous patients. Interestingly, ASD and PDA were both present in our patient #1, a previously undescribed coexistence. Similarly, anomalies as left hemihypertrophy and supernumerary nipples observed in patient #1, and short neck, microcornea, nystagmus, and hypertrophic cardiomyopathy, occurring in patient #2, have not been previously observed in OES (Table 1), expanding thus the disease's clinical spectrum. The genetic etiology of OES was recently identified through comparative whole‐genome sequencing in DNA from a variety of tissues of unrelated affected individuals (Boppudi et al., 2016; Peacock et al., 2015). Using this approach, *KRAS *somatic mutations were demonstrated, including c.38G>A (p.Gly13Asp) and c.57G>C (p.Leu19Phe) (Peacock et al., 2015). More recently, three additional patients with OES were shown to carry the *KRAS* codon 146 variants c.436G>A (p.Ala146Thr) and c.437C>T (p.Ala146Val) (Boppudi et al., 2016). In the present work, we molecularly examined two OES patients (#1 and #2) and identified the two previously reported *KRAS *codon 146 mutations. Such pathogenic variants were firstly reported as cancer‐associated somatic mutations, particularly in colorectal cancer (CRC), but have been also found in other malignancies such as multiple myeloma, pancreatic ductal adenocarcinoma, and non‐small cell lung cancer (Feig & Cooper, 1988). Mutations in *KRAS *codon 146 seem to be associated with improved overall survival of patients with CRC compared to subjects carrying other *KRAS *variants (Janakiraman et al., 2010).

ECCL is a distinct uncommon oculo‐neuro‐cutaneous disease with a prevalence of <1/1,000,000 (https://www.orpha.net/consor/cgi-bin/OC_Exp.php?Lng=EN&Expert=2396) which was first described by Haberlans and Perou in a subject with severe mental retardation, epilepsy, unilateral choristoma, and cutaneous lesions, and central nervous system abnormalities (Haberland & Perou, 1970). ECCL exhibits an extensive and variable clinical spectrum, but cardinal major and minor criteria have been already defined (Hunter, 2006; Moog, 2009). In our patient #3, an ECCL diagnosis was supported by the involvement of two systems classified with major criteria (eye choristoma and nevus psiloliparus) and at least one minor skin criteria (skin tags); in the patient #6, the diagnosis was supported by the involvement of three systems, with major criteria in ≥2 (choristoma, small skin tag, skin hypoplasia, arachnoid cyst, and cortical atrophy). Nevus psiloliparus and choristomas are observed in up to 80% of ECCL patients, whereas skin tags are another common sign that can be identified in up to 70% of cases (Moog, 2009). Optic nerve coloboma, identified in the right eye of our ECCL patient (#6), has been previously reported in at least two subjects with the disease (Chandravanhi, 2014; Hunter, 2006). Somatic *FGFR1 *and *KRAS *mutations were recently demonstrated in subjects with ECCL (Bennett et al., 2016; Boppudi et al., 2016). FGFRs belong to the RTKs (receptor tyrosine kinases) family of proteins which regulate a range of functions including cell growth differentiation/proliferation, tissue patterning, and organogenesis (Dorey & Amaya, 2010). The c.1638C>A (p.Asn546Lys) *FGFR1 *mutation has been recurrently identified in ECCL cases (Bennett et al., 2016) while one patient with clinical criteria for both OES and ECCL was found to carry the c.436G>A (p.Ala146Thr) *KRAS *mutation (Boppudi et al., 2016). Thus, the identification of the *FGFR1* p.Asn546Lys variant in our ECCL patient #3 confirms this missense change as the major cause of ECCL. On the other hand, the c.437A>G (p.Ala146Val) *KRAS* variant detected in our ECCL patient #6 is a new mutation for ECCL, although other point mutations at the same codon has been previously associated with the disease (Boppudi et al., 2016).

In 1895, Jadassohn first described a patient with a congenital cutaneous lesion of linear distribution, which was initially classified as naevi sebacei, and subsequently as nevus sebaceous of Jadassohn (Jadassohn, 1895; Robinson, 1932). In 1957, Schimmelpenning recognized the association of such cutaneous lesion with brain, eye, and skeletal anomalies (Schimmelpenning, 1957), and shortly after Feuerstein and Mims reported two cases with the clinical triad of linear nevus sebaceous with epilepsy and mental retardation (Feuerstein & Mims, 1962). The estimated incidence of nevus sebaceous of Jadassohn is around 1–3 per 1,000 live birth, but its incidence is unknown when it is associated with systemic abnormalities as in the SFMS (Warrenburg, Gulik, Renier, Lammens, & Doelman, 1998). Up to 60% of SFMS patients have brain abnormalities, particularly hemimegalencephly, ventriculomegaly, and cortical atrophy (Lantis, Leyden, Thew, & Heaton, 1968; Lin & Yan, 2010). Consistently, in our SFMS patients #4 and #5 brain defects were demonstrated, including uncommonly reported anomalies such as polymicrogyria (Clancy et al., 1985) or arachnoid cyst (Kamate Dumale & Hattiholi, 2009). Contrarily, intellectual disability, a finding in up to 70% of SFMS cases (Warrenburg et al., 1998), was absent in our two SFMS probands. Eye abnormalities can be recognized in ~60% of patients with SMFS and both of our SFMS patients exhibited eye choristoma, which is the most commonly identified ocular anomaly; optic nerve coloboma, a defect observed only in 7% of patients with this syndrome (Arevalo, Lasave, Arevalo, & Shields, 2013; Paulidis, Cantalupo, Boria, Cossu, & Pisani, 2012; Shields, Shields, Eagle, Arevalo, & DePotter, 1997), was also observed in our patients. Bilateral hearing loss, identified in SFMS patient #4, has been previously described unilaterally in a single subject (Gowdar, Nyamagoudar, & Chezhian, 2015). Our SFMS patients exhibited the linear cutaneous lesions on the scalp and face, which are the most common localization (Eisen & Michael^2009^). Interestingly, our SFMS patient #5 had a pattern of mucocutaneous hyperpigmentation reminiscent of that observed in Peutz‐Jeghers syndrome patients (Beggs et al., 2010).

Previously, mutations in *KRAS*, *HRAS*, and *NRAS *genes have been reported in patients with isolated nevus sebaceous of Jadassohn or SFMS (Groesser et al., 2012; Igawa et al., 2016; Levinsohn et al., 2013; Lihua et al., 2017; Peacock et al., 2015; Sun et al., 2013; Wang et al., 2015). To our knowledge, only 7 patients with SFMS have been molecularly analyzed to date (Table 2) and identified mutations include *HRAS *c.37G>C (p.Gly13Arg) in three patients (Groesser et al., 2012; Lihua et al., 2017; Sun et al., 2013), *KRAS *c.35G>A (p.Gly12Asp) in two subjects, *KRAS *c.34G>T (p.Gly12Cys) in a single patient (Groesser et al., 2012; Igawa et al., 2016), and *NRAS *c.182 A>G (p.Gln81Arg) in another patient (Kuroda et al., 2015). In our study, two SFMS subjects (patients #4 and #5) were demonstrated to be mosaics for the *KRAS* c.35G>A (p.Gly12Asp) mutation, which can be now considered the most common disease pathogenic variant.

The identification of somatic mutations in *KRAS* and *FGFR1* genes in patients with cutaneous mosaic disorders could provide a tool for early diagnosis and surveillance of associated disorders. Non‐ossifying fibromas resulting in long bones stress fractures (Ardinger et al., 2007; Boppudi et al., 2016; Fickie & Stoler, 2011; Mermer, Kayhan, Karacelebi, & Percin, 2016; Peacock et al., 2015), giant cell granuloma of the mandibule and maxilla (Ardinger et al., 2007; Boppudi et al., 2016; Federici et al., 2004; Mermer et al., 2016; Peacock et al., 2015; Toriello et al., 1999), rhabdomyosarcoma (Ardinger et al., 2007), craniofacial, intracranial or intraspinal lipomas (Hunter, 2006), jaw osteomas (Zielinska‐Kazmierska, Grodecka, Jablonska‐Polakowska, & Arkuszewski, 2005), odontomas (Andreadis, Rizos, Belazi, Peneva, & Antoniades, 2004; Hauber, Warmuth‐Metz, Rose, Bröcker, & Hamm, 2003), ossifying fibromas (Moog et al., 2007), pilocytic‐low grade astrocytoma (Bieser et al., 2015; Brassesco et al., 2010; Valera et al., 2012), papillary glioneural tumor (Phi et al., 2010), dysembryoplastic neuroepithelial tumor (Bavle et al., 2018), and optic glioma (Kocak et al., 2016) can occur in these three oculocutaneous syndromes. In addition, malignant secondary tumors can arise from the nevi sebaceous in SFMS patients, including basal cell carcinoma and squamous carcinoma (Eisen & Michael^2009^, ^2009^; Idriss & Elston, 2014). Primary malignant tumors in SFMS can include Wilm's tumor, nephroblastoma, salivary gland adenocarcinoma, stomach and esophagus carcinoma, and astrocytoma, among others (Berkeley, 1959; Eisen & Michael^2009^),

Finally, as part of our study, genetic screening of *KRAS*, *HRAS*, *NRAS *and *FGFR1 *genes was carried out on DNA isolated from epibulbar dermoid tissue from two patients without syndromic disease. No pathogenic variants were identified in these genes suggesting that other loci from the same or from a different molecular pathway are involved in the isolated development of this ocular tumor. Alternatively, a very low level of mosaicism for a pathogenic mutation, unidentifiable by the employed method, could be responsible for this anomaly.

In conclusion, we have studied a cohort on Mexican patients with oculocutaneous mosaic RASopathies. Our results support the inclusion of molecular analysis of *KRAS *and *FGFR1 *genes as part of major criteria of OES, ECCL, and SFMS. The knowledge of the molecular cause has important implications for prognosis and therapy in patients suffering from such mosaic developmental syndromes.

## CONFLICT OF INTEREST

The authors declare no potential conflict of interests.
